# Dr. James Taylor

**Published:** 1842-12

**Authors:** 


*Dr. James Taylor.*—This gentleman, so long and extensively known in Kentucky and Mississippi, as a scientific and skilful practitioner of Dental Surgery, has recently located himself in Cincinnati, Ohio. There are few men in the profession better qualified to exercise its duties, than Dr. Taylor, and his abilities will hardly fail to be appreciated in the Queen City.

